# Improved Method of Blockchain Cross-Chain Consensus Algorithm Based on Weighted PBFT

**DOI:** 10.1155/2022/5169259

**Published:** 2022-08-25

**Authors:** Liu Lei, Liangtu Song, Jiahua Wan

**Affiliations:** ^1^Institute of Intelligent Machines, Hefei Institutes of Physical Science, Chinese Academy of Sciences, Hefei 230031, China; ^2^University of Science and Technology of China, Hefei 230026, China; ^3^Anhui Xinhua University, Hefei 230088, China

## Abstract

Aiming to solve the problems of low fault tolerance, low throughput, and high delay in traditional methods, an improved method of the blockchain cross-chain consensus algorithm based on weighted PBFT is proposed. This article constructs a blockchain cross-chain exchange model based on cluster centers and divides the nodes in the blockchain system into consensus service nodes, cross-chain exchange nodes, and application nodes to improve the performance of consensus computing services. On this basis, according to the weighted PBFT consensus mechanism, the blockchain consensus environment is set up, and the distribution of nodes in the consensus domain and the blockchain signature scheme are obtained. Therefore, the blockchain cross-chain consensus optimization algorithm is designed to reduce throughput and delay and optimize the consensus effect. The experimental results show that the proposed method can effectively improve the shortcomings of traditional methods, with high throughput and low latency, and strong security. It shows that it is a low resource consumption and secure consensus method.

## 1. Introduction

Blockchain is a new technology integrated with distributed storage, peer-to-peer (P2P) networking, consistency verification, consensus algorithm, cryptography, and other computer technologies [1]. It uses blockchain data structure to verify and store data, uses a consensus algorithm to generate and update data, uses cryptography to ensure the security of data transmission and access, uses an intelligent contract composed of automatic script code to program and operate data, and realizes trusted data management in the incomplete trusted environment [2]. Amongst them, the consensus algorithm is the core part of the blockchain, which directly affects the efficiency, security, and stability of the whole system. At this stage, blockchain is accelerating the development of the digital economy and is deeply integrated with real industries. However, selecting or designing an appropriate blockchain cross-chain consensus algorithm according to business needs is difficult for researchers and developers [3, 4].

Under the above background, reference [5] proposed a master-slave multichain blockchain consensus mechanism based on reputation, designed a two-layer blockchain structure to build a master-slave multichain mechanism, and connected multiple chains through the main blockchain. From the blockchain, the global consistency of digital assets is guaranteed, and the performance of the blockchain is improved. A reputation evaluation is introduced into the consensus mechanism based on proof of rights and interests, and a joint consensus mechanism integrating multiple consensus mechanisms is designed to ensure data consistency and tamper-proof modification. By generating dynamic verification nodes, the decentralization of nodes is ensured, and malicious attacks are prevented. The simulation experiment results show that this method has the advantage of high security in dealing with right smashing attacks and bribery attacks, but it has the problem of weak fault tolerance. Reference [6] proposed an efficient blockchain consensus algorithm based on directed acyclic graphs. The algorithm uses the directed acyclic graph data structure based on ID classification, which can reach a consensus more simply, and is suitable for multiple users to confirm transactions simultaneously. The experimental results show that the consensus algorithm can save a large amount of hardware resources and improve the transaction processing effect of the blockchain, but it has the problem of low throughput. At the system operation level, an adaptive controller together with fractional-order parameter adaptation laws is designed based on combining the parallel distributed compensation technique and the fractional Lyapunov stability theory to guarantee the Mittag-Leffler stability in the closed-loop system [7]. Reference [8] presents a composite learning fuzzy control to synchronize two different uncertain incommensurate fractional-order time-varying delayed chaotic systems with unknown external disturbances and mismatched parametric uncertainties via the Takagi-Sugeno fuzzy method. An adaptive controller together with fractional-order composite learning laws is designed based on both a parallel distributed compensation technology and a fractional Lyapunov criterion. The boundedness of all variables in the closed-loop system and the Mittag-Leffler stability of tracking error can be guaranteed. Reference [9] proposed a high-reliability blockchain consensus mechanism based on contribution value and difficulty value. According to the node's contribution value ranking, the nodes are assigned the corresponding proof of work (PoW) difficulty value, and the nodes then compete for the accounting rights through the PoW consensus mechanism. The consensus mechanism after the introduction of PoW respects the proof of capacity (PoC) contribution value ranking to the greatest extent. Thus, the node's accounting block rate is highly proportional to its contribution value. At the system operation level, the accounting block rate is guaranteed to reach or infinitely approach 100%, which effectively solves the problem of system suspension caused by PoC. The PoW difficulty value distribution algorithm is designed from the perspectives of the node contribution value ranking, the value difference between adjacent contribution value nodes, and the grouping method. The rationality and the effectiveness of the difficulty value distribution algorithm are verified through experiments. The superiority and the feasibility of this scheme are verified through experiments, but when the nodes are many, this method has a certain time delay, and the real-time performance is poor.

The above discussion shows that despite many blockchain formulas proposed by related scholars, certain problems remain, such as excessive delay and low throughput. Each consensus algorithm has problems, which is also one of the main issues restricting the development of blockchain. Consensus algorithms are an important part of the blockchain. Improving the consensus algorithm in the blockchain is the most important link in improving the performance of the blockchain. Therefore, starting from improving throughput, reducing delay, and improving fault tolerance performance, this article proposes an improved method of the blockchain cross-chain consensus algorithm based on weighted PBFT and improves the blockchain consensus algorithm according to the blockchain use scenario.

## 2. Blockchain Cross-Chain Exchange Model Based on Cluster Centre

**Figure 1:**
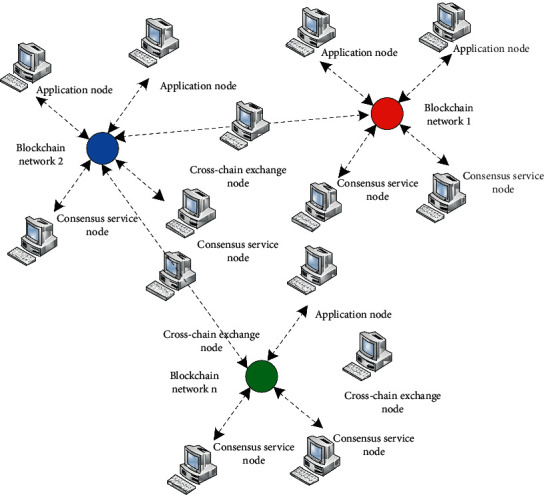
Schematic diagram of blockchain cross-chain exchange model.

In view of the existing problems of blockchain systems, this article proposes a blockchain cross-chain exchange model based on cluster centers. This model divides the nodes in the consortium blockchain system into three different types of nodes: consensus service nodes, cross-chain exchange nodes, and application nodes [10]. The consensus service nodes with efficient computing capability through a high-speed network are connected to form a blockchain P2P network serving a business field [11], which specifically provides consensus computing services for the application nodes in the blockchain network. The cross-chain switching node is connected to different blockchain networks at the same time, the block data of different blockchains are synchronized, the status database of different blockchain networks on this node is formed, and a blockchain switching network between switching nodes based on the P2P protocol is formed to provide cross-chain access services for application nodes of different blockchain networks. The application node can synchronize data from its blockchain network consensus service node, access cross-chain exchange nodes, and send intrachain or cross-chain transactions. The schematic diagram of the blockchain cross-chain exchange model is shown in Figure 1.

In Figure 1, the blockchain network is divided into different specialized blockchain networks according to service or application fields and businesses; different blockchain networks have different requirements for service capacity and transaction efficiency, and they have corresponding calculations. Computing nodes that are capable and can connect to the main blockchain network at a high speed are used as consensus service nodes that provide consensus computing services to the blockchain network application nodes through computing power competition.

In the blockchain cross-chain exchange model, the collection of transaction nodes is denoted as *D*, the single node is denoted by *d*_*i*_, and the transaction data are denoted by *s*_*d*_. The consensus node collection is denoted as *A*, the nodes in the set are numbered as {0,1,…, *n* − 1}, and a single node is denoted as *a*_*i*_. The collection of data storage nodes is denoted by *E*, and a single node is denoted by *e*_*i*_.

Each node *d*_*i*_ in *D* sends a transaction *α*, which is propagated to the consensus node set *A* through the P2P network. In the absence of intermediate nodes and because the consensus nodes do not trust one another, the nodes must exchange information and verify one another to reach a consensus [12]. If the transaction data sent by a certain consensus node *A*_*ij*_ to other nodes are *s*_*ij*_, the transaction received by this node from other nodes is represented by a vector *G*_*t*_(*t*_0_, *t*_1_,…, *t*_*n*_), (*t*_0_, *t*_1_, .., *t*_*n*_) is a set of vectors for transactions of different nodes, and the modulus of the vector is |*G*_*t*_| ≤ |*D*| − 1.

A function *f*(*x*) and an algorithm (protocol/process) *Y* are designed such that

(1) $$\begin{array}{c} G_{t}=f\left(t_{0},t_{1},\ldots ,t_{n}\right),\\ \left\{\begin{array}{cc} \text{Store}, & Y\left(t\right)=\mathrm{ture,}\\ \mathrm{Discard,} & Y\left(t\right)=\mathrm{false}. \end{array}\right) \end{array}$$

The final consensus result obtained through the transaction vector received by function *f*(*x*) and consensus node *A*_*ij*_ is equal to the original transaction information. After a transaction of the algorithm *Y*, if it is approved by most nodes, the algorithm result is TRUE, and the “Store” instruction is sent to the data storage node. If it is FALSE, the “Discard” instruction is executed and current transaction data are discarded. The model is essentially a data pipeline, and transaction data flow between different types of nodes.

Malicious nodes are in the consensus node, deliberately tampering with transaction data, causing the problem of “double consumption.” Therefore, the consensus nodes are divided into three categories, namely, *A*_1_, *A*_2_, and *A*_3_. The first two are honest nodes, and the latter is a malicious node. If *A*_1_ receives *A*_2_'s transaction vector as *∂*_*A*_, and the transaction vector received by *A*_3_ is *∂*_*A*′_, then function *f*(*x*) and algorithm *Y* must meet the following conditions:

(2) $$\begin{array}{c} \left\{\begin{array}{c} \partial_{A^{\prime }}=\frac{f\left(\partial_{A^{\prime }},\partial_{A}\right)}{a_{i}},\\ \text{Store}\left(\partial_{A}\right)\neq \text{True.} \end{array}\right) \end{array}$$

Node *A*_*ij*_ can identify which transactions are illegal and which transactions are legal without intermediate nodes, and store the legal transactions as the execution result in the blockchain database. Illegal transactions cannot be executed at any time.

## 3. Blockchain Cross-Chain Consensus Algorithm Optimization

### 3.1. PBFT Consensus Mechanism

The consensus mechanism layer is responsible for the data consistency amongst the nodes in the whole blockchain system network. The data of all nodes in the blockchain are stored independently. Therefore, a mechanism is needed to ensure that the ledger data stored by each node of the blockchain are consistent, and the role of the consensus layer is to ensure that the information on the chain is transparent and the data can be shared [13].

**Figure 2:**
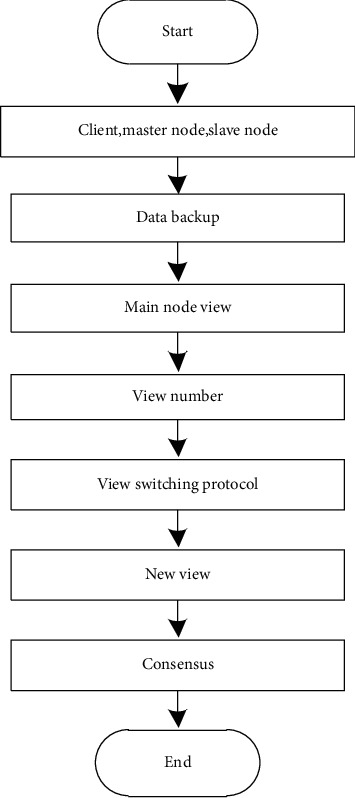
PBFT consensus flow chart.

PBFT is an algorithm used to solve the Byzantine Generals problem, which can ensure consistency between nodes in the presence of malicious nodes in the network. The PBFT algorithm has three roles: client, master node, and slave node. The master node and the slave node perform data backup. During PBFT, the consensus process led by a master node is in one view [14]. The view is the definition of the node relationship in the PBFT consensus mechanism, and the number of the view is marked as *Z*. In a view, different nodes have different numbers, and each view has only one main node. In the event of a failure of the master node and timeout of the consensus process, the master node needs to be switched according to the view switching protocol in the PBFT consensus mechanism to generate a new view *Z*′ and continue the consensus process in this view.  Figure 2 shows the PBFT consensus flow chart.

The specific PBFT consensus steps are as follows:

1. Request stage: The client sends a request 〈Request:*r*, *c*, *v*〉.
2. Prepreparation phase: The master node assigns a number *M* to the received request and broadcasts a prepreparation message in the format pre〈*R* : *r*, *c*, *v*, *p*〉, where *p* is the digest value of the request, which is generated using a secure hash function.
3. Preparation stage. The node that receives the prepreparation message verifies *p* in the prepreparation message and enters the preparation phase if the verification passes. The preparation phase broadcasts the preparation message. The format is 〈*R:r,c,l*〉, and *l* is the number of the node itself. The pre〈*R:r,c,v,p*〉 and 〈*R:r,c,v*〉 messages are written to the log whilst broadcasting. When the master node receives the pre〈*R:r,c,v,p*〉 and 〈*R:r,c,v*〉 messages from *N*+1 different nodes and passes the verification, the preparation phase is completed, and the main verifications are *r*, *c*, and *v*.
4. Confirmation stage. After the node completes the preparation phase, it enters the confirmation phase and broadcasts the confirmation message in the format 〈Comment:*r,c,k*_*f*_*,l*〉, where *k*_*f*_ is the signature set of the slave node. When the 〈Comment:*r,c,k*_*f*_*,l*〉 message is confirmed, *k*_*f*_ and *l* are mainly verified. When *N*+1 confirmation messages including themselves are received and verified, the confirmation phase is completed.
5. Recovery stage. The response format is 〈Reply:*r,c,p,l,h*〉, where *h* is the execution result of the request. When the client receives the same request result from *N*+1 different nodes, it believes that the network has reached this consensus.

### 3.2. Blockchain Cross-Chain Consensus Algorithm Optimization

According to the weighted PBFT consensus mechanism, the blockchain consensus environment is set up, and the distribution of nodes in the consensus domain is obtained, thereby designing the blockchain cross-chain consensus optimization algorithm to reduce throughput and delay and optimize the consensus effect.

#### 3.2.1. Blockchain Consensus Environment

**Figure 3:**
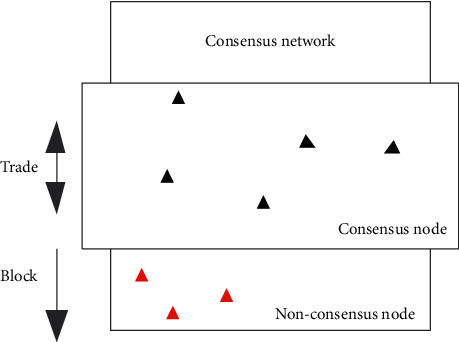
Blockchain consensus environment.

Transactions can be created and propagated on any node of the alliance chain. Blocks are created by consensus nodes and propagated to other nodes. The verification and processing rules of transactions and block data are governed by the Ethereum protocol; consensus data are propagated on the consensus network by consensus mechanism constraints. The data flow between nodes is shown in Figure 3.

In the alliance chain network, the set of alliance chain nodes is *B*, its size is *U*_0_, the set of consensus nodes is *B*^*η*^, its size is *U*_1_, the set of nonconsensus nodes is *B*^*μ*^, and its size is *U*_2_, satisfying

(3) $$\begin{array}{c} \left\{\begin{array}{c} U_{0}=U_{1}\cup U_{2},\\ U_{1}\cap U_{2}=\emptyset . \end{array}\right) \end{array}$$

The nodes in set *B* are numbered as (*b*_1_, *b*_2_,…, *b*_*t*_), and the single node is numbered as *ω* is denoted as *B*_*ω*_ [15], which satisfies

(4) $$\begin{array}{c} \forall B_{\omega }\in B\left(B^{\eta }+B^{\mu }\right), \end{array}$$

and at the same time meets

(5) $$\begin{array}{c} B\in B^{\eta };\\ B\in B^{\mu }. \end{array}$$

The consensus network may have multiple error nodes. The set of consensus nodes is *B*^*η*^, and the set of error nodes is *B*^*τ*^, *i* is the weight coefficient in the node-set *m*, and *j* is the weight coefficient in the node set *n*, which satisfies

(6) $$\begin{array}{c} B\geq \sum_{i=1}^{m}\sum_{j=1}^{n}{\left[B_{ij}^{\eta }-B_{ij}^{\tau }\right]}^{2}. \end{array}$$

In the blockchain consensus environment based on the Byzantine fault-tolerant mechanism, if the number of error nodes that exist can meet formula (6), the Byzantine General problem can be solved, the fault tolerance is good, and the correctness and activity are strictly proven. In practice, the number of consensus nodes is at least 4.

#### 3.2.2. Distribution of Consensus Domain Nodes

**Figure 4:**
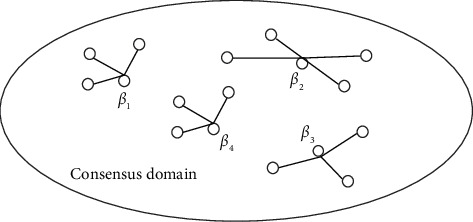
Schematic diagram of node distribution.

In the blockchain consensus environment, all consensus nodes in the consensus domain are divided into four groups, namely, *β*_1_, *β*_2_, *β*_3_, and *β*_4_, and each group member has a different weight in the consensus process, as shown in Figure 4.

Given that the voting weight of group *β*_1_ members is *W*_1_=1, the weight of group *β*_2_ is *W*_2_=2, the weight of group *β*_3_ is *W*_3_=3, and the weight of group *β*_4_ is *W*_4_=4. The members of group *β*_4_ have the highest voting weight and believe that they are the most secure and least prone to evil nodes. Through this division, the security of the consensus network depends more on those groups with high voting weight [16].

The Byzantine decision criterion undergoes several changes because nodes have different voting weights. In traditional algorithms, the voting weights of nodes are the same, which can be considered 1. Assuming that the total number of nodes in the entire network is *N*′, the maximum number of malicious nodes allowed in the consensus network is *C*, and the Byzantine judgment criterion requires *N*′=3*C*+1. In the new consensus environment, the weighted sum of votes of the entire network is defined as *ρ*_*N*′_, and the weighted sum of the maximum allowable votes of malicious nodes as *W*_*f*_. Then, *ρ*_*N*′_ is calculated as:

(7) $$\begin{array}{c} \rho_{N^{\prime }}=\frac{\rho_{l}\varphi_{l}\left(T_{i}-T_{j}\right)}{T_{W}}. \end{array}$$

Amongst them, *ρ*_*l*_ represents the weighted similarity value, *φ*_*l*_ represents the trustworthiness, *T*_*i*_ represents the density of secure nodes, *T*_*j*_ represents the density of malicious nodes, and *T*_*W*_ represents the total weight of the vote.

*W*_*f*_ is calculated as:

(8) $$\begin{array}{c} W_{f}=\int_{T_{f}}^{T_{W}}c_{ij}\left(T_{i}-T_{j}\right)d\left(T_{i}-T_{j}\right). \end{array}$$

Amongst them, *c*_*ij*_ represents the average trust degree of the node.

When all nodes belong to group *β*_4_, the algorithm becomes the traditional PBFT consensus algorithm.

The weighted PBFT optimization algorithm is explained based on the PBFT algorithm. All consensus nodes are divided into main domain nodes and subdomain nodes. The nodes of group *β*_4_ constitute the main domain, and the nodes of group *β*_1_, *β*_2_, and *β*_3_ constitute the subdomain [17]. The number of nodes in the main domain is defined as *β*_4_(*N*), and the upper limit of the malicious nodes that can be accommodated in the main domain is *W*_*f*_(max).

The selection rule of the master node is to select from the main domain randomly but this is different from the traditional algorithm, which is randomly selected from the entire network. This has the advantage of reducing the frequency of master node changes. The switch of the master node causes a view switch. To ensure consistency, a large amount of additional communication is required. Therefore, the switch of the master node reduces consensus efficiency. Given that the security level of the nodes in the main domain is the highest, the probability of the master node's error is low because the view switching caused by the master node's error occurs less frequently, which reduces consensus overhead and improves consensus efficiency.

#### 3.2.3. Blockchain Signature Scheme

The consensus node is required to sign the block because the traditional PBFT algorithm does not verify whether the received block comes from a legal consensus node. The specific rules are as follows:

1. When creating a proposal block, the consensus node list is used as the block header data to participate in the calculation of the block hash value to ensure that the set of nodes participating in each round of consensus negotiation cannot be tampered with.
2. When the proposed block is submitted to the chain, the cache list consisting of the consensus node index and the signature of the block is synchronously submitted. Although the cache list of different nodes may be different, the number of list elements strictly meets the vote requirement.
3. When synchronizing the history and adding a new block, the sender node broadcasts the block whilst sending the list cache list, and the receiver node verifies it. If the signature verification passes, the block is uploaded to the chain [18].

#### 3.2.4. Realisation of Blockchain Cross-Chain Consensus

When traditional algorithms execute consensus protocols, a large amount of communication occurs between nodes. As the number of nodes and transactions increases, network communication increases rapidly, which increases bandwidth pressure and affects the consensus efficiency of the algorithm. Therefore, this article aims to solve this problem, adopts the weighted PBFT algorithm, combined with the characteristics of the alliance chain, and in the absence of Byzantine nodes, optimizes the consensus protocol to reduce the amount of communication between nodes. Furthermore, the integration mechanism and the elevator mechanism are introduced, such that the algorithm can quickly restore to the optimal state when Byzantine nodes appear in the network and execute the optimized consensus protocol most of the time [19].

The weighted PBFT algorithm is an improvement on the PBFT algorithm. It also completes the consensus operation through mutual communication between nodes in the network, and its communication is executed according to the consensus protocol. The algorithm in this article improves the consensus protocol of the PBFT algorithm and designs an optimized consensus protocol to reduce the amount of communication between nodes in the consensus process.

The specific execution of the weighted PBFT algorithm is as follows:

(1) Initialize the node. First, the nodes in the network are numbered, and the points of the nodes are initialized to 100 points[20]. Second, the consensus node set *B*^*η*^ and the candidate node set *B*^*λ*^ are initialized.
  (9) $$\begin{array}{c} B^{\lambda }=\left\{1,2,3,\ldots N-1\right\}. \end{array}$$
Then, the number of consensus nodes and candidate nodes is
  (10) $$\begin{array}{c} \left\{\begin{array}{c} \left|B^{\eta }\right|=N_{f^{\prime }}^{2},\\ \left|B^{\lambda }\right|=N_{d^{\prime }}^{2}. \end{array}\right) \end{array}$$
In (10), *f*′ represents the consensus weight of consensus nodes. *d*′ represents the expected weight of the candidate node candidate set.
(2) The client sends a transaction request to the master node. After the master node receives the request, the request message is numbered, and then the master node executes the optimized consistency protocol.
(3) All consensus nodes execute the optimized consistency protocol. In the confirmation phase of the optimized consistency protocol, the state of the consensus node is judged. At this stage, the master node receives the feedback messages from all consensus nodes, judges the correctness of the feedback messages, compares them with the locally saved prepreparation messages, and judges whether the corresponding values of the fields are the same. Once the transaction information is tampered with, its hash value changes. Therefore, two comparison results appear in the system, and the algorithm performs different operations according to different comparison results [21].
(4) The master node upgrades the node according to the integration of the node, and updates the consensus node set and candidate node set to ensure that the high probability of consensus nodes are honest nodes, and then continues to implement the optimized consistency protocol in the next consensus process [22]. In summary, the use of the weighted PBFT algorithm to improve blockchain cross-chain consensus is completed.

## 4. Simulation Experiment

In order to verify the effectiveness of the blockchain cross-chain consistency algorithm based on weighted PBFT, simulation and comparison experiments are carried out. It is compared with the master-slave multichain blockchain consensus mechanism based on reputation and the efficient blockchain consensus algorithm based on a directed acyclic graph. Next, they are compared and evaluated in terms of fault tolerance, throughput, and delay. This test is conducted in a CloudSim cloud computing environment, and the network bandwidth is 80 mi S^−1^, 8 GB memory, 10 servers, and SPSS simulation software is used to process the experimental data.

### 4.1. Experimental Indicators

1. Throughput refers to the number of network nodes that the algorithm can carry after being used in the system.
2. Fault tolerance refers to the tolerance value of the consensus algorithm to nodes that have non-Byzantine faults and the tolerance value of nodes that have Byzantine faults in the system. Fault tolerance is also one of the references for algorithm security.
3. Latency mainly reflects the efficiency of the consensus algorithm in an application.
4. Safety: Consensus algorithm safety mainly refers to malicious behavior incidence. In the current complex network environment, ensuring the security and stable operation of the blockchain is the focus of the current research.

### 4.2. Analysis of Experimental Results

**Experiment 1 .**

**Figure 5:**
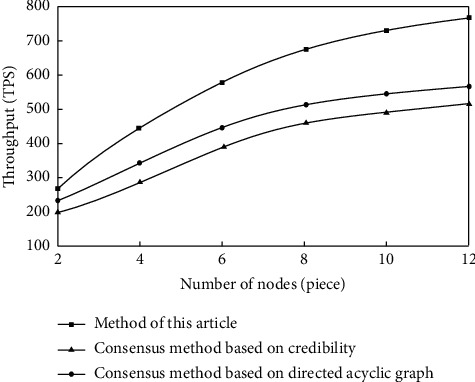
Throughput comparison results.

To obtain transaction data better, the transaction simulation module is introduced into the blockchain system. Through the newly built 100 simulated transaction accounts, to make the transaction sustainable, the initial quota of each transaction account is 10000, and the volume of each transaction is 1 to ensure that the transaction can occur at the fastest speed and test the throughput of the consensus algorithm. The throughput comparison results of the three methods are shown in Figure 5.

According to the analysis of Figure 5, with the increase in the number of nodes in the network, the throughputs of the three methods show an upward trend, but overall, the throughput of the method in this article is much higher than that of the master-slave multichain blockchain consensus mechanism based on reputation and the efficient blockchain consensus algorithm based on the directed acyclic graph because the traditional method suspends the consensus process for a period of time, during which the algorithm only completes the consensus work of one transaction, which affects the throughput of the algorithm. This method does not have this problem. Thus, the throughput is substantially higher than that of the two traditional methods.

**Experiment 2 .**

Comparing the fault tolerance of different methods, the results are shown in Table 1, where *n* represents the total number of nodes in the system.

Table 1 shows that this method can tolerate that the proportion of non-Byzantine error nodes is *n*/2, with high consistency, high availability, and strong antifraud ability. The fault tolerance of the master-slave multichain blockchain consensus mechanism based on reputation is *n*/3, and any attempt to destroy the system entails a large amount of costs that outweighs the losses. The fault tolerance of the efficient blockchain consensus algorithm based on the directed acyclic graph is *n*/5, and the security is poor. The comparison shows that the fault tolerance of this method is good, which shows that it has high security.

**Experiment 3 .**

To verify the effectiveness of the method in this article further, the time delay is taken as the experimental index to compare the application effects of different methods. The results are shown in Table 2.

Table 2 shows that in the case of relatively few transaction nodes, the three methods spend almost the same time, and the method in this article has a slight advantage. However, with the increase in the number of transaction nodes, the advantage of the method in this article is gradually evident, which is far from the time spent by the traditional method. This method has great advantages in transaction efficiency and is suitable for the application scenario of large-scale blockchain deployment.

**Experiment 4 .**

**Figure 6:**
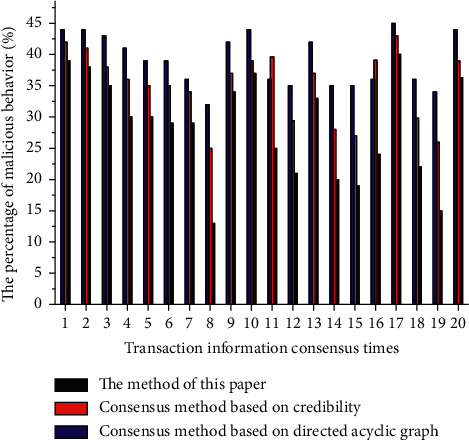
Safety contrast.

In order to further verify the security of the consensus algorithm designed in this article, the security of the three methods is compared with the proportion of malicious behaviors as the experimental indicator. Given the same system parameters, 20 transaction information consensuses were performed for the three consensus algorithms. The occurrence of malicious behaviors in the system is recorded separately, and then the proportion of malicious behaviors in the consensus algorithm is statistically compared. The experimental results are shown in Figure 6.

As can be seen from Figure 6, the average malicious behaviors are 28.47%, 35%, and 39.1%, respectively, of this method. As can be seen from the experimental results, this article has significant advantages in reducing the probability of malicious behavior in the system, effectively improving system security.

## 5. Conclusion

Summarizing the advantages and disadvantages of traditional methods, to improve the effect of blockchain consensus further, a method for improving the blockchain cross-chain consensus algorithm based on weighted PBFT is proposed. A blockchain cross-chain exchange model based on a cluster center is established, the blockchain consensus environment is set, the distribution of consensus domain nodes and the blockchain signature scheme is obtained, and the blockchain cross-chain consensus optimization algorithm is designed. The experimental results show that the proposed method has higher throughput, lower latency, and stronger security, which fully verifies the effectiveness of the method. It is expected to provide a reference for researchers and developers when selecting or innovatively designing consensus algorithms and to promote the evolution of blockchain consensus algorithms.

## Figures and Tables

**Table 1:** Comparison results of fault tolerance.

Method	Fault tolerance
Method of this article	*n*/2
Master-slave multichain blockchain consensus mechanism based on reputation	*n*/3
Efficient blockchain consensus algorithm based on directed acyclic graph	*n*/5

**Table 2:** Delay comparison results.

Number of transaction nodes/piece	Method of this article	Consensus mechanism of master-slave multichain blockchain based on reputation	Efficient blockchain consensus algorithm based on the directed acyclic graph
4	2.5	2.6	2.6
8	2.8	2.8	3.0
12	3.2	3.2	3.4
16	3.5	4.1	4.7
20	3.6	4.5	5.0
24	3.7	4.9	5.5
28	3.8	5.2	6.0
32	3.9	5.7	6.6

## Data Availability

The data used to support the findings of the study are available from the corresponding author upon request.
